# Extraction, Characterisation and Evaluation of Antioxidant and Probiotic Growth Potential of Water-Soluble Polysaccharides from *Ulva rigida* Macroalgae

**DOI:** 10.3390/foods13111630

**Published:** 2024-05-23

**Authors:** Naraporn Phomkaivon, Preeyanut Pongponpai, Prapat Kosawatpat, Bussaba Thongdang, Wanida Pan-utai

**Affiliations:** 1 Department of Food Chemistry and Physics, Institute of Food Research and Product Development, Kasetsart University, Bangkok 10900, Thailand; ifrnpph@ku.ac.th; 2 Faculty of Science, Prince of Songkla University, Songkhla 90110, Thailand; preeyanut1415@gmail.com; 3 Phetchaburi Coastal Aquaculture Research and Development Center, Coastal Aquaculture Research and Development Division, Department of Fisheries, Phetchaburi 76100, Thailand; prapat1120@gmail.com (P.K.); nok.annann@gmail.com (B.T.); 4 Department of Applied Microbiology, Institute of Food Research and Product Development, Kasetsart University, Bangkok 10900, Thailand

**Keywords:** *Ulva*, macroalgae, water extraction, polysaccharide, antioxidant, monosaccharide, probiotic

## Abstract

*Ulva rigida* green macroalgae contain a variety of polysaccharides. A recent study investigated the optimum concentration and yield of polysaccharide extraction from oven-dried *U. rigida* biomass using a water-soluble polysaccharide extraction method that adhered to safety standards. This study utilised complete factorial experiments to examine the effects of varying factors on polysaccharide extraction. Results showed a positive correlation between increased levels of all factors and higher polysaccharide extraction yield. This study also found that the main factors and their interaction had a significant impact on the extracted polysaccharides from *U. rigida*. The highest polysaccharide content and yield were 9.5 mg/mL and 189 mg/g, respectively. Water-soluble polysaccharides demonstrated the presence of reducing sugar (8 mg/g), phenolics (0.69 mg/g) and flavonoids (1.42 mg/g) and exhibited antioxidant properties. Results revealed that freeze-dried polysaccharide powders were primarily composed of the monosaccharide rhamnose. Preliminary results on the effect of these powders on probiotics demonstrated that supplementation of polysaccharides from *U. rigida* promoted viable *Lactobacillus rhamnosus* ATCC 53103 growth during cultivation. This discovery has the potential to revolutionise the human food industry and promote the development of functional ingredients for novel and future food products, with numerous applications in the nutraceutical and pharmaceutical industries.

## 1. Introduction

Modern consumers are more aware of their well-being, with increasing interest in functional foods. There has been growing awareness of the importance of promoting healthy and sustainable diets as one of the critical strategies to safeguard human and environmental health [1]. Functional foods can be produced by enriching traditional foods with bioactive compounds. These compounds can be sourced from various materials including plants, macroalgae and microalgae and have demonstrated significant health benefits. However, the incorporation of these compounds into functional food formulations requires the extraction of the desired compounds. In the food industry, there is a growing preference for natural products, emphasising product safety, quality and functionality, as well as utilising environmentally friendly techniques that avoid harmful solvents. To achieve these goals, researchers and industry practitioners are exploring various methods of extraction that eliminate the need for toxic solvents [2]. This approach ensures that the production process is environmentally sustainable while maintaining the final product’s safety and quality [3]. 

Seaweeds, also referred to as macroalgae, are categorised based on their chlorophyll nature, cell wall chemistry and flagella presence [2]. These marine plants are a rich and abundant source of biologically active phytochemicals, which include carotenoids, phycobilins, fatty acids, polysaccharides, vitamins, sterols, tocopherols and phycocyanins [4]. Phytochemicals show potential for use in healthcare due to their remarkable biological activity. However, the nutritional and chemical composition of seaweeds can vary based on factors such as species, geographical origin, seasonal and environmental fluctuations, harvest time, water temperature and processing techniques [5]. These factors must be considered when evaluating the potential use of seaweeds as a source of beneficial phytochemicals. Macroalgae are known to possess a significant quantity of carbohydrates accumulated within their cells. These carbohydrates, including monosaccharide, oligosaccharide and polysaccharide, provide the human body with ample energy and support various physiological functions and physical activities. Essential carbohydrates cannot be substituted with alternative energy sources [6]. Green macroalgae are rich in polysaccharides, which are of considerable interest [7].

The *Ulva* species of macroalgae exhibit a cell wall composition primarily constituted by polysaccharides, accounting for approximately 18% of dry weight [8]. Methods for polysaccharide extraction from cells include hot water, acidic and alkaline conditions and mechanical-assisted techniques through microwave, ultrasonic and enzymatic hydrolysis [9]. Due to its high polarity, non-toxicity, safety in production and ability to effectively penetrate plant tissues, water is the favoured solvent for extracting polysaccharides. The conventional approach for extracting polysaccharides involves hot-water extraction [10]. Recent investigations have revealed that *Ulva* polysaccharides have diverse physiological properties such as antioxidant, anticoagulant, antitumour, anti-ageing and immune regulatory activities [11]. These findings have been substantiated in various scientific studies, further highlighting the immense potential of polysaccharides from *Ulva* spp. as chemical agents and medicines in the medical and agricultural industries [12,13]. Polysaccharides derived from macroalgae are considered functional prebiotics. These polysaccharides cannot be easily digested by the human gastrointestinal tract but they can be utilised by the gut microbiota to generate metabolites and produce several beneficial effects [14]. Polysaccharides from macroalgae exhibit the potential to support probiotic growth in the host gut for balanced health and can be applied in healthy food and ingredients [15]. 

The objective of this study was to investigate the maximum polysaccharides extracted from oven-dried *U. rigida* green macroalgae using full factorial experiments. The water-soluble extract under various extraction conditions was evaluated for polysaccharides, reducing sugar, phenolics and flavonoid contents and antioxidant capacities. Freeze-dried polysaccharides extracted from *U. rigida* were determined for monosaccharide composition and their potential to promote bacteria as a prebiotic substance.

## 2. Materials and Methods

### 2.1. Macroalgae Preparation

Green macroalgae *Ulva rigida* C. Agardh or Sea Lettuce were collected from the Phetchaburi Coastal Aquacultural Research and Development Center, Coastal Aquaculture Research and Development Division, Department of Fisheries, Thailand [16]. *U. rigida* algae were cultured in seawater, with salinity controlled at 30–32 ppm. The maximum *U. rigida* biomass was harvested after 21 days of cultivation and washed with fresh water to remove the residual seawater [17]. Fresh macroalgae were dried in a hot air oven, with the temperature controlled at 60 °C for 6 h. The oven-dried biomass preparation was milled to 0.5 mm size and stored in a polyethylene bag in the dark at room temperature. 

### 2.2. Proximate Composition

The chemical composition of *U. rigida* oven-dried biomass was analysed according to AOAC standard methods [18]. Crude protein content was determined using the Kjeldahl method and crude lipid was extracted via Soxhlet extraction using petroleum ether. The resulting lipid was dried until constant weight. Crude fibre content was assessed using both acid and alkaline digestion methods, with the fibre residue also dried until constant weight. Ash content was analysed by igniting dried samples in an electric furnace at 550 °C. Finally, carbohydrate content was calculated by subtracting the sum of moisture, protein, lipid, fibre and ash contents from 100 g of dry matter.

### 2.3. Polysaccharide Extraction Experiments

**Figure 1:**
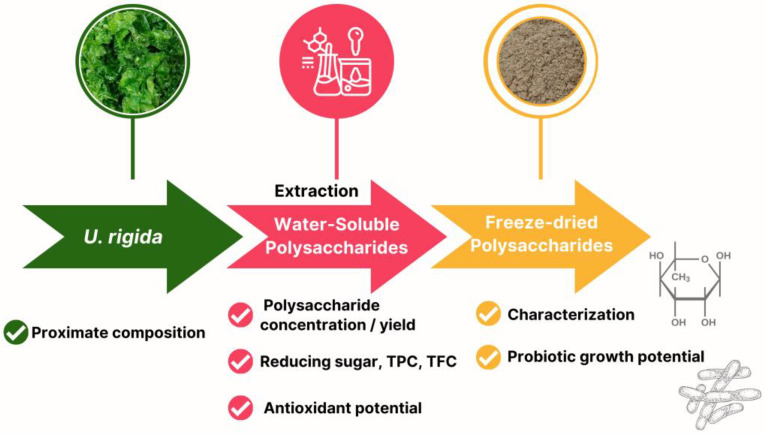
Schematic diagram outlining the process of extracting water-soluble polysaccharides from *U. rigida* macroalgae, followed by characterisation and evaluation of freeze-dried polysaccharides for their probiotic growth potential.

Water-soluble polysaccharide extraction from *U. rigida* was performed using a two-level full factorial design with four factors to optimise the significant conditions for maximum polysaccharide extraction. These factors included biomass–solvent ratio, extraction temperature, extraction time and cycle of extraction (Table 1). The *U. rigida* biomass was mixed with deionised (DI) water, with the extraction temperature controlled in a water bath (WNB10, Memmert, Schwabach, Germany). The mixture was hand-mixed at 10 min intervals and the water-soluble polysaccharides extracted were separated using a centrifuge at 3660× *g* for 20 min (Frontier^TM^ 2000 Multi Centrifuges, Ohaus, Parsippany, NJ, USA). DI water was added to the biomass pellet residue for the second extraction cycle, and the water-soluble polysaccharides extracted from *U. rigida* were subjected to further analysis. Moreover, the schematic diagram clearly illustrates the workflow in Figure 1.

### 2.4. Analysis 

#### 2.4.1. Determination of Polysaccharides

Polysaccharide content was determined via the phenol–sulphuric method according to Wang, et al. [19] with minor modification. First, 0.5 mL of water-soluble polysaccharides extracted from *U. rigida* was placed in a test tube. Then, 5% phenol was added, and the mixture was thoroughly mixed using a vortex mixer. After that, 2.5 mL of sulphuric acid was added, and the mixture was incubated at room temperature for 20 min. Finally, the samples were measured at 490 nm using a spectrophotometer (SP-8001, UV-Vis Spectrophotometer, Metertech, Taipei, Taiwan) with glucose used as the standard. The polysaccharide content was calculated and expressed as glucose in mg/mL (*C_PS_*). Polysaccharide extraction yield (*yield*) was calculated with Equation (1) and expressed as milligrams per gram of dried biomass (mg/g).

(1) $$Yield\;\left(mg/g\right)=\frac{C_{PS}\times V}{Biomass}$$

where $C_{PS}$ is the concentration of polysaccharide (mg/mL), V is the DI solvent extraction (mL) and Biomass is the dried biomass (g).

#### 2.4.2. Determination of Reducing Sugar

The reducing sugars present were analysed using the dinitrosalicylic acid (DNS) method following Miller (1959) [20] with some modifications. A sample of 0.25 mL was mixed with 0.5 mL of DI water and 0.25 mL of the DNS reagent. This mixture was then boiled in a water bath for 15 min and left to cool in cold water to stop the reaction. After that, 2 mL of DI water was added and the absorbance of the solution was measured at 540 nm using a spectrophotometer (SP-8001, UV-Vis Spectrophotometer, Metertech, Taiwan). Glucose was used as the standard for reducing sugar and expressed as glucose in milligrams per gram of dried biomass (mg/g).

#### 2.4.3. Total Phenolic Content

Total phenolic content (TPC) was measured with the Folin–Ciocalteu method, as described by Pan-utai et al. [17]. Briefly, 20 µL of the sample or standard was mixed with 10% Folin–Ciocalteu reagent (100 µL) in a 96-well plate and the mixture was incubated in the dark at room temperature for 8 min. After that, 80 µL of 7.5% sodium carbonate and 50 µL of DI water were added and the resulting mixture was thoroughly mixed and incubated at 40 °C for 30 min. The absorbance was measured at 750 nm using a microplate reader (M965+, Microplate Reader, Metertech, Taiwan), and gallic acid was used as the standard. The TPC was expressed as milligram gallic acid equivalent per gram of dried biomass (mg GAE/g).

#### 2.4.4. Total Flavonoid Content

Total flavonoid content (TFC) was determined according to the method of Liu et al. [21] with some modifications. A 100 µL aliquot of sample or standard was mixed with an equal amount of aluminium chloride solution. The mixture was then incubated at room temperature for 10 min and the absorbance was measured at 405 nm using a microplate reader (M965+, Microplate Reader, Metertech, Taiwan). Quercetin was used as the standard. The TFC was expressed as milligram quercetin equivalent per gram of dried biomass (mg QE/g).

#### 2.4.5. Antioxidant Activity via DPPH Assay

The DPPH radical-scavenging activity assay was performed following Hung, et al. [22] with minor modifications. In brief, a 100 µL sample or standard was combined with 100 µL of 200 µM DPPH solution (2,2-diphenyl-1-picrylhydrazyl, Sigma-Aldrich, Singapore). After 30 min incubation in the absence of light at room temperature, the absorbance was measured at 750 nm using a microplate reader (M965+, Microplate Reader, Metertech, Taiwan). Ascorbic acid was used as the standard. The antioxidant capacity was expressed as mg ascorbic acid equivalent per gram of dried biomass (mg AAE/g).

#### 2.4.6. Antioxidant Activity via ABTS Assay

The ABTS radical-scavenging activity assay was conducted following the method described by Pan-utai et al. [17]. In brief, 245 mM ammonium persulphate at 505.05 and 5.05 µL, respectively, and 7 mM ABTS (22′-azino-bis (3-ethylbenzothiazoline-6-sulphonic acid) diammonium salt were mixed to create the ABTS radical solution, and the combination was left to incubate for 16 h at ambient temperature in the dark. The prepared solution was diluted with DI water to provide 0.7 absorbance at 750 nm. Afterwards, 190 µL of ABTS solution was combined with 10 µL of either the sample or the standard on a 96-well plate and incubated for 5 min at room temperature in the dark. A microplate reader (M965+, Microplate Reader, Metertech, Taiwan) was used to measure the absorbance at 750 nm, with ascorbic acid used as a standard. The amount of antioxidant capacity per gram of dried biomass was reported as mg ascorbic acid equivalent (mg AAE/g).

#### 2.4.7. Antioxidant Activity via FRAP Assay

The ferric-reducing antioxidant power (FRAP) assay was conducted following the method described by Pan-utai et al. [17]. In summary, the reagent was made by mixing 10 mM TPTZ (2,4,6-tris(2-pyridyl)-s-triazine) with 300 mM sodium acetate (pH 3.6) in 40 mM HCl and 20 mM ferric chloride at 25, 2.5 and 2.5 mL, respectively. After that, 190 µL of FRAP reagent was combined with 10 µL of either the sample or the standard in a 96-well plate and incubated at room temperature for 30 min in the dark. The absorbance was measured using a microplate reader at 593 nm (M965+, Microplate Reader, Metertech, Taiwan), with ascorbic acid used as a standard. The antioxidant capacity was expressed as mg AAE/g or milligrams of ascorbic acid equivalent.

### 2.5. Monosaccharide Determination

Water-soluble polysaccharides from *U. rigida* were prepared under the optimum extraction condition using a biomass–solvent ratio of 1:20 (*w*/*v*) and an extraction temperature of 90 °C for 2 h. The supernatant was separated via centrifugation at 3660× *g* for 20 min. The extracted polysaccharides were then stored in a freezer at −30 °C for 18–24 h, followed by freeze-drying 30–60 Pa for 38 h. The resulting freeze-dried polysaccharides from *U. rigida* were used to determine monosaccharides as the substrate to evaluate their effect on probiotics.

Monosaccharide analysis was performed using HPAEC-PAD. The freeze-dried polysaccharides extracted from *U. rigida* were dissolved in ultrapure water (18 MΩ-cm, Merck Millipore, Darmstadt, Germany) at 2 mg/mL concentration. For acid hydrolysis, 200 μL of sample was mixed with 200 μL of 0.2 N trifluoroacetic acid (TFA) for 2 h at 100 °C following the method described by Weitzhandler, et al. [23] with some modifications. The mixture was evaporated under pressure until dryness and then redissolved with deionised water. Both samples were measured via high-performance anion exchange chromatography (HPAEC) using a pulsed amperometric detector (PAD). The system (Dionex™ ICS 5000, Thermo Scientific, Vacaville, CA, USA) was equipped with a CarboPac™ PA20 column (6 µm particle size, 3 × 150 mm) and CarboPac™ PA20 guard column (6 µm particle size, 3 × 30 mm). The isocratic of 16 mM sodium hydroxide at a flow rate of 0.4 mL/min was conducted for 20 min. The injection volume was 5 μl and the column temperature was set at 30 °C. The waveform “standard carbohydrate quad” and a pH-Ag/AgCl reference electrode were used. Six monosaccharides including arabinose, galactose, glucose, fructose, rhamnose and xylose (Sigma-Aldrich, Singapore) were used as standards, with data processed using Chromeleon™ 6.8 Chromatography Data System software.

### 2.6. Effect of Probiotic

The procedure was modified from the method presented by Song, et al. [24] to analyse the impact of polysaccharides from *U. rigida* on probiotic growth. *Lactobacillus rhamnosus* ATCC 53103 was used as the reference bacterial strain for the probiotics. First, *L. rhamnosus* ATCC 53103 was prepared in MRS broth and incubated at 37 °C for 18 h. Then, 10% (*v*/*v*) of *L. rhamnosus* ATCC 53103 was transferred to MRS broth and incubated at 37 °C for another 12 h. The cells were centrifuged at 3660× *g* for 10 min, washed twice with 0.1% peptone and resuspended in 0.1% peptone. The cells were then diluted to an absorbance of 0.2 at 600 nm to prepare the probiotic bacterial suspension. Next, a *L. rhamnosus* ATCC 53103 bacterial suspension of 10% (*v*/*v*) was inoculated into minimum medium broth supplemented with 2% (*w*/*v*) freeze-dried polysaccharides from *U. rigida*, while glucose and inulin were utilised as the controls. The bacterial mixture was cultured at 37 °C for 48 h and samples were collected at 0, 6, 12, 24 and 48 h to evaluate the viable cells of *L. rhamnosus* ATCC 53103 using the plate-count method on MRS agar.

### 2.7. Statistical Analysis

Results were shown as mean values ± standard deviation (SD) for each experiment run in triplicate, with statistical analysis carried out utilising SPSS version 25.0 (IBM Corp., Armonk, NY, USA). The effects of the four parameters—biomass–solvent ratio, extraction temperature, extraction duration and extraction cycle—on polysaccharide content and extraction yield were assessed using two levels of full factorial design. All experiment parameters were compared using Duncan’s multiple range test (DMRT) at a significance level of 0.05.

## 3. Results

*Ulva rigida* macroalgae are a significant source of polysaccharides which have various applications. Water is a safe solvent that shows promise for extracting bioactive compounds. To achieve the maximum concentration and yield of polysaccharides, oven-dried *U. rigida* biomass was extracted using water. The optimum extraction condition was determined using a two-level complete factorial design of essential factors. Various assays were conducted to determine the reducing sugar, total phenolic and flavonoid contents, as well as antioxidant capacities. The water-soluble polysaccharides extracted were also found to support bacterial growth.

### 3.1. Proximate Composition

Table 2 presents the chemical composition of oven-dried *U. rigida* macroalgae. The proximate composition analysis revealed that green *Ulva* species primarily comprised polysaccharides with high carbohydrate content.

### 3.2. Water-Soluble Polysaccharide Extraction

This study analysed polysaccharide concentration (C_PS_) and extraction yield from *U. rigida*. Various factors were taken into account including biomass–solvent ratio (ratio), extraction temperature (temp) and time (time) and cycles of extraction (cycle). Analyses of the main effects and interactions between polysaccharide concentration and extraction yield at various conditions are shown in Table 3. Results indicated that all dependent variables significantly differed between the main factors and interaction (*p* < 0.05), except for the yield based on the biomass–solvent ratio. C_PS_ and yield showed no significant interactions between extraction temperature and time, extraction time and cycle, biomass–solvent ratio and temperature and time, biomass–solvent ratio and extraction time and cycle and also biomass–solvent ratio and extraction temperature and time and cycle. Therefore, biomass–solvent ratio, extraction temperature and time and extraction cycle affected C_PS_ and yield.

Table 4 shows multiple comparisons between the various extraction conditions. C_PS_ and yield under various conditions ranged from 0.18 to 7.28 mg/mL and from 7.27 to 159.23 mg/g, respectively. A biomass–solvent ratio of 1:20 (*w*/*v*), extraction temperature at 90 °C for 2 h and the first extraction cycle gave the highest water-soluble polysaccharide concentration of 7.28 mg/mL, whereas the highest extraction yield was over 159 mg/g from a biomass–solvent ratio 1:40 (*w*/*v*) at 90 °C for 2 h in the first extraction cycle. Among the different conditions, polysaccharide content and yield in the first cycle were higher than in the second cycle.

Figures S1–S3 display the concentrations and yields of water-soluble polysaccharides extracted from *U. rigida* under various conditions. Results showed that longer extraction time led to higher polysaccharide concentration and extraction yield compared to different biomass–solvent ratios (Figure S1). Therefore, polysaccharide extraction was most efficient at higher extraction temperatures. Figure S2 illustrates the correlation between polysaccharide concentration and extraction yield at different temperatures. Higher biomass concentration led to higher polysaccharide concentrations at various temperature extractions (Figure S2a,c). Extraction yield at 70 °C showed no significant difference between different biomass concentrations, while the highest yield was obtained from the lower biomass concentration at 90 °C (Figure S2b,d). The correlation between extraction times is demonstrated in Figure S3. At higher biomass concentrations for different extraction times, higher concentrations of polysaccharides were observed. Conversely, when lower biomass concentrations were utilised during different extraction times, higher extraction yields were noted.

Table 5 presents polysaccharide concentrations and extraction yields obtained under different conditions. The highest concentration of polysaccharides (9.45 mg/mL) was achieved after two extraction cycles with a biomass–solvent ratio of 1:20 (*w*/*v*) at 9 °C for 2 h. The highest extraction yields obtained at biomass–solvent ratios of 1:20 and 1:40 (*w*/*v*) were not significantly different (189 mg/g and 187 mg/g, respectively). The optimum extraction temperature was 90 °C for 2 h, with higher polysaccharide concentrations and yields obtained at longer extraction times and higher temperatures. The highest polysaccharide concentration and extraction yield were obtained from the highest biomass–solvent ratio (1:20% *w*/*v*), at an extraction temperature of 90 °C, with an extraction time of 2 h and only one cycle. Water-soluble polysaccharides extracted from *U. rigida* macroalgae at optimum conditions were prepared and used as the freeze-dried polysaccharide preparation for monosaccharide composition and probiotic growth determination.

### 3.3. Reducing Sugar, Total Phenolic and Flavonoid Contents

*U. rigida* water-soluble extracts contain non-reducing polysaccharides and reducing mono or oligosaccharides. Table 6 presents the results of testing different polysaccharide conditions using soluble extraction to determine reducing sugar contents. The first extraction cycle at a biomass concentration ratio of 1:20 (*w*/*v*) yielded the highest amount of reducing sugar, while only the first extraction cycle resulted in reducing sugar when the biomass–solvent ratio was 1:40 (*w*/*v*). Highest reducing sugar yields of 7.95 mg/g and 6.58 mg/g at ratios of 1:20 and 1:40 (*w*/*v*), respectively, were obtained at an extraction temperature of 90 °C for 2 h.

Water-soluble polysaccharides extracted from *U. rigida* have various compositions. To determine the TPC and TFC, the extraction was carried out using different conditions, as shown in Table 6. TPC was obtained from the first cycle of extraction using two levels of biomass–solvent ratio, extraction temperature and time. TPC was not detected in all the experiments from the second extraction cycle. TPC ranged between 0.18 and 0.69 mg GAE/g, while TFC ranged from 0.02 to 1.42 mg QE/g under various conditions. The highest TPC and TFC values of 0.69 mg GAE/g and 1.42 mg QE/g were obtained from the highest biomass concentration, extraction temperature and time of 1:20 (*w*/*v*) at 90 °C and 2 h, respectively.

### 3.4. Antioxidant

Antioxidant properties of polysaccharides extracted from *U. rigida* were evaluated using DPPH, ABTS and FRAP assays under different conditions, as shown in Table 7. Water-soluble polysaccharides extracted at various conditions exhibited a DPPH radical-scavenging capacity ranging from 0.02 to 10.49 mg AAE/g. The highest DPPH was achieved at 70 °C for 1 h from a lower biomass concentration of 1:40 (*w*/*v*). ABTS radical-scavenging activity at different conditions ranged from 0.11 to 0.94 mg AAE/g. Polysaccharides extracted under various conditions were determined using the ferric reducing antioxidant power (FRAP) assay, with results ranging from 0.17 to 0.52 mg AAE/g. The antioxidant properties showed a variety of value trends from various antioxidant assays. The water-soluble polysaccharides extracted from *U. rigida* were a greenish colour visible to the naked eye, indicating high total phenolic and flavonoid contents and antioxidant capacities.

### 3.5. Monosaccharide Composition

Freeze-dried polysaccharides extracted from *U. rigida* included numerous monosaccharides. Six monosaccharides were separated using HPAEC-PAD analysis between 6.507 and 12.217 min. Each calibration curve was plotted using linear regression calculations. The correlation coefficient (R²) values ranged between 0.992 and 0.999 (Table S1). Six standards were used to identify the analyte peak area of the sample with acceptable accuracy.

Table 8 shows the monosaccharide content of water-soluble polysaccharides obtained from *U. rigida* acid hydrolysis of polysaccharides. The acid hydrolysed sample consisted of rhamnose as the significant component at 78.97 mg/g followed by xylose, glucose and galactose, with arabinose as the minor component at 0.33 mg/g sample. By contrast, only a small amount of glucose was detected in the polysaccharide extract. The low level of monosaccharides in the polysaccharide extract suggested that the main composition was oligosaccharides or long-chain polysaccharides composed of rhamnose, xylose and glucose.

## 4. Discussion

Green macroalgae, also known as seaweed, are a promising source of biochemicals with various nutritional benefits. They contain polysaccharides ranging from 15 to 76%, proteins ranging from 5 to 47%, minerals ranging from 7 to 36% and lipids ranging from 1 to 5% [25]. Seaweed has been used in traditional medicine for centuries and is considered a sustainable and eco-friendly source of nutrition. The potential health benefits of seaweed make it an attractive option for the food and pharmaceutical industries. Macroalgae are an excellent biomass source rich in various bioactive polysaccharides, peptides, polyphenols, pigments and other bioactive compounds, which have potential applications in the food industry and biomedical sectors as functional ingredients [26,27]. A significant proportion of carbohydrates was obtained from *U. rigida*, accounting for 48% of the dry biomass. Our results were consistent with the high carbohydrate content typically found in macroalgae, with one study reporting the carbohydrate content found in different samples of *U. rigida* ranging from 16.63 to 65.93% of dry biomass [28]. Another study found that the carbohydrate content in *U. fenestrate* varied between 25 and 38% of dry biomass, with the highest amount obtained under elevated irradiance and temperature conditions [29]. Environmental factors such as temperature, irradiance, nutrients, CO_2_ levels and season significantly impact carbohydrate content and other biochemical compositions in microalgae [30,31]. The carbohydrates present in macroalgae mainly consist of polysaccharides, along with a small quantity of oligosaccharides and monosaccharides [32]. Macroalgae represent a cost-effective source of valuable and beneficial compounds [33].

Various methods have been applied for the extraction of bioactive compounds [7]. Hot-water extraction is the most reliable and effective technique for extracting bioactive compounds from algae, particularly polysaccharides [34]. Water, a safe and non-toxic solvent, can easily penetrate plant tissue, making hot-water extraction the conventional method for polysaccharide extraction [10]. Despite the challenges posed by algal cell walls, our results identified crucial factors for optimising extraction conditions to enhance the extraction of valuable macroalgal polysaccharides. The extraction of water-soluble polysaccharides from *U. rigida* was influenced by various extraction factors such as ratio, temperature, time and cycle. To achieve the highest extraction yield and bioactive compound extraction, both individual and combined extraction conditions were studied [35]. The solvent-to-biomass ratio is one of the factors that impact polysaccharide extraction. The evaluation of significant factors showed that increasing the biomass–solvent ratio led to high polysaccharide solubility in water extraction. Extraction temperature and time also impacted polysaccharide extraction. Higher extraction temperatures led to a greater recovery of bioactive compounds from *U. lactuca* [36]. Polysaccharide extraction from algae has been performed using several methods. Table 9 compares polysaccharide extraction yields for various algal species and solvents, highlighting the different methods employed. Among multiple species and extraction methods, our results showed a polysaccharide yield of 18.9% as the optimum result. Extraction methods affect algal yield which also depends on algal species, cultivation methods, season and environmental conditions [7,37]. The data obtained indicate that higher extraction temperatures lead to a significant increase in polysaccharide yields, as a result of enhanced dissolution [10]. One of the main advantages of this extraction method is that it is simple and cost-effective [38]. According to the findings in Table 9, hot-water extraction at 90 °C for 2 h provides a significantly higher yield compared to using an 80% ethanol solution at 73 °C for 2 h and 6 min. Additionally, it also provides a superior yield compared to other hot-water extraction techniques (ranging from 80 °C to 97 °C) with a shorter duration. This method of extraction is not only environmentally friendly but also energy-saving and solvent-free. It is considered safe for food production and significantly reduces the need for solvent and acid separation from the polysaccharides. Furthermore, polysaccharide extracted using hot water tends to have an original profile compared to acidic solvents, which can cause acid hydrolysis during the extraction process.

Water-soluble extracts from macroalgae release polysaccharides and also other bioactive substances. Monosaccharides are reducing sugars containing hemiacetal or hemiketal groups, while polysaccharides are non-reducing. During water-soluble extraction, the reducing sugar in the supernatant extracted from *U. rigida* was maximised at 8 mg/g. A previous study reported maximum reducing sugar extracted from *U. prolifera* biomass of 0.156 g/g using a thermochemical method at 0.9 M H_2_SO_4_ and 121 °C for 50 min [46]. Enzymatic hydrolysis of *U. fasciata* macroalgae yielded maximum reducing sugar levels of 1.61 g/L at 6 h hydrolysis time [47]. Reducing sugar production in *Gracilaria verrucosa* macroalgae was achieved via ultrasonication with an acid catalyst followed by enzymatic hydrolysis [48]. Maximum reducing sugar content was achieved with extreme extraction or hydrolyses at high temperatures using acidic solutions and mechanical-assisted techniques. Our results found that glucose was expressed as a reducing sugar during polysaccharide extraction under non-extreme conditions with a mild solvent such as water. Macroalgae contain secondary metabolites that form phenolic and flavonoid compounds and are recognised for their antioxidant properties and essential functions in numerous biological processes [49,50]. Our results determined similar total phenolic and flavonoid contents as found in previous studies. In *U. lactuca* green macroalgae and *Laurencia obtuse* red macroalgae, increasing the biomass–solvent ratio increased the total phenolic compound content [36,51]. A methanolic extraction of *U. clathrate* and *U. intestinalis* from the Persian Gulf found that flavonoid contents varied from 8 to 33 mg RE/g [52]. Chemical constituents and phenolic and flavonoid contents from macroalgae vary depending on the season, environment and extraction conditions. The macroalga *U. rigida* demonstrated excellent antioxidant properties with DPPH, ABTS and FRAP assays. Green macroalgae possess noteworthy antioxidant potential, which could be applied in several industries including medicine, cosmetics, dietary supplements and food [52]. This discovery has significant implications for various applications in these sectors. Our results suggested that green macroalgae are a valuable source of antioxidants for multiple applications and warrant further investigation. These findings expand the current knowledge base and can be shared with external stakeholders. Polysaccharides extracted from green macroalgae exhibited antioxidant properties. They can be used as antioxidant ingredients in the food industry. Furthermore, their potential as a hepatoprotective agent in the pharmaceutical industry has also been demonstrated [12].

The carbohydrates present in macroalgae are composed of different sugar monomers [53], with extraction methods and conditions significantly impacting the yield, composition and structure of the resultant product. Changes in the bioactivity of polysaccharides impact the quality and efficacy of the final product [10]. Various monosaccharides were reported in polysaccharides from *Ulva* spp., with macroalgae including glucose, rhamnose, arabinose, xylose, mannose, galactose, fucose, glucosamine and glucuronic acid [8,54,55]. The monosaccharide composition and ratio in polysaccharides from *Ulva* spp. varied depending on species, growth conditions and harvesting season [4,56]. Among sixblade species and one filamentous species (Table 10), rhamnose (10.90–257.00 mg/g) was the significant component in *Ulva*, followed by glucose (3.60–183.10 mg/g) and xylose (4.40–99.00 mg/g) [57,58,59,60]. Galactose was detected at 0.42–28.27 mg/g [57,58,59,60]. Mannose was found in *U. meridionalis* (filamentous) and *U. ohnoi* as a minor component [60]. *U. rigida* from Chile contained arabinose, fucose and fructose in small amounts, while these monosaccharides were not detected in *U. rigida* from New Zealand. Our results indicated that the pattern of monosaccharides in *U. rigida* from different sources was mainly composed of rhamnose, xylose and glucose. The complexity of monosaccharide components, glycosidic linkage and sulphated group substitution of *Ulva* polysaccharides contributed to their biological antioxidant activity and immunomodulatory and hypolipidemic activity, as well as increasing the proliferation of probiotics in the intestinal microbiome [8,58,61]. Furthermore, the antioxidant activity of *Ulva* polysaccharides is significantly correlated with molecular weight. Notably, the degraded polysaccharide from *U. pertusa* Kjellm with a low molecular weight of 28.2 kDa demonstrated substantially higher scavenging activity for superoxide and hydroxyl radicals, as well as the strongest reducing power and metal chelating activity, in comparison to higher molecular weight variants (58.0–151.7 kDa) [62]. *Ulva* polysaccharides are endowed with unique attributes including hydrogels, nanofibers, 2D structures and 3D porous structures, rendering them an ideal option for drug delivery and tissue engineering applications. Their capacity to effectively stabilise and release drugs or selectively target bioactive compounds in hydrophilic matrix systems makes them particularly promising for a range of biotechnological applications [63,64]. In addition, rhamnose-rich oligosaccharides and polysaccharides are potential bioactive compounds that can serve as functional food ingredients and also in the pharmacological and medical fields [4,8,65].

**Figure 2:**
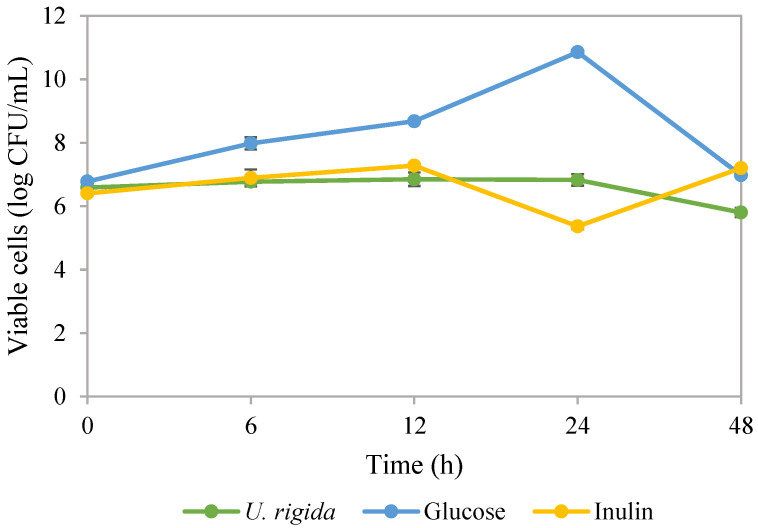
Viable cells of *Lactobacillus rhamnosus* ATCC 53103 under different supplemented ingredients in culture broth.

The consumption of probiotics can improve the health of the host. However, ensuring the survival of these probiotics in challenging environments is difficult. One promising solution is using polysaccharides, which are non-toxic, highly compatible with biological systems and biodegradable. These polysaccharides can form a protective layer around the probiotics, providing a physical barrier and aiding in their delivery, thereby making them a potential solution for probiotic delivery [67]. During the growth of *Lactobacillus rhamnosus* ATCC 53103 probiotic bacteria, freeze-dried polysaccharides extracted from *U. rigida* were replaced as a substrate in the culture medium. Results showed that viable *L. rhamnosus* ATCC 53103 cells could grow for up to 48 h, with a maximum of 6.85 log CFU/mL (Figure 2). The control medium contained glucose with maximum viable cells of 10.86 log CFU/mL. Inulin, a commercial prebiotic, showed similar cell growth compared to polysaccharides from *U. rigida*, with maximum cell growth of 7.2 log CFU/mL. Polysaccharides possess the ability to act as prebiotics, meaning they can influence the composition of the gut microbiome, promote gut barrier health and regulate the production of metabolites by the gut microbiota [68]. Prebiotics improve immunity, resist pathogens, influence metabolism, increase mineral absorption and enhance health [69]. Our results are in concordance with a previous study that used ulvan polysaccharide as a prebiotic in yoghurt products [70]. Our findings suggest that *L. rhamnosus* probiotic, when promoted with polysaccharides from *U. rigida*, has the potential to be effective in a similar way to previous reports. Polysaccharides such as laminaran, porphyran and ulvan extracted from brown, red and green macroalgae were found to promote the growth of *L. rhanmosus* ATCC 53103, *L. plantarum* ATCC 10241 and *Bifidobacterium breve* ATCC 15700 [71]. Our research findings suggest that the polysaccharides present in *U. rigida* can act as carbon sources to promote cell growth. These polysaccharides also possess hydrolysis enzymes that are not present in the human genome [68]. Polysaccharides found in green macroalgae consist of sulphated L-rhamnose and D-glucoronic acid. The whole genome sequence of *L. rhamnosus* ATCC 53103 suggests that certain enzymes are involved in the hydrolysis process, with gene GCF_000011045.1_00051 being responsible for hydrolysis enzymes that play a role in β-glucosidase, α-L-rhamnosidase and β-glucan 1,4-α-glucosidase [71,72]. This study investigated the feasibility of utilising polysaccharides derived from *U. rigida* to support bacterial growth. Results suggested that these polysaccharides could enhance the viability and functionality of probiotic microorganisms, thus contributing to the quality and safety of food products. These findings underscore the potential value of this novel approach for improving the quality and safety of food products and advancing our understanding of the interplay between polysaccharides, beneficial bacteria and human health. Polysaccharides extracted from *U. lactuca,* incorporated with a yoghurt formulation at 1–2%, were reported to have good physicochemical properties, as well as stimulating the growth and activity of probiotic bacteria comprising *Lactobacillus acidophilus*, *Streptococcus thermophilus* and *Bifidobacterium* sp. [70]. Moreover, the combination of macroalgae-derived polysaccharides with probiotics can be used as dietary supplements to enhance the health of aquatic animals [73].

## 5. Conclusions

*U. rigida* C. Agardh green macroalgae contain significant amounts of polysaccharides. This study evaluated the maximum concentration and yield of a safe water-soluble polysaccharide extraction from oven-dried *U. rigida* biomass using full factorial experiments. Increasing all factors resulted in higher levels of polysaccharides extracted. The main factors and their interaction had a significant effect on the extracted polysaccharides from *U. rigida*. The water-soluble polysaccharides contained reducing sugar, phenolics and flavonoids and exhibited antioxidant activities. Freeze-dried polysaccharide powders mainly consist of the monosaccharide rhamnose. Preliminary results on the effect of these powders on probiotics suggested that when supplemented with polysaccharides from *U. rigida*, viable *L. rhamnosus* ATCC 53103 growth was promoted during cultivation. This discovery has the potential to impact the human food industry through the development of functional ingredients for novel and future food products, with various other applications in the nutraceutical and pharmaceutical industries.

## Figures and Tables

**Table 1:** Two-level full factorial design experiments for water-soluble polysaccharide extraction from *U. rigida* macroalgae.

Factor	Levels	
Biomass-solvent ratio (*w*/*v*)	1:20	1:40
Extraction temperature (°C)	70	90
Extraction time (h)	1	2
Number of extraction cycles	1	2

**Table 2:** Proximate composition of *U. rigida* oven-dried macroalgae.

Composition	% Dry Weight
Crude protein	24.29 ± 0.29
Crude lipid	0.62 ± 0.09
Crude fibre	6.12 ± 0.01
Ash	20.84 ± 0.26
Carbohydrate	48.14 ± 0.48

**Table 3:** Main effects and interactions of polysaccharide concentration and extraction yield from *U. rigida* macroalgae under various conditions.

Factor	Dependent Variable	*SS*	*df*	*MS*	F-Value	*p*-Value
Ratio	C_PS_	53.464	1	53.464	1385.158	0.000 ^a^
	Yield	68.032	1	68.032	2.064	0.157
Temp	C_PS_	19.465	1	19.465	504.299	0.000 ^a^
	Yield	15,363.200	1	15,363.200	466.001	0.000 ^a^
Time	C_PS_	5.706	1	5.706	147.822	0.000 ^a^
	Yield	3357.666	1	3357.666	101.846	0.000 ^a^
Cycle	C_PS_	174.699	1	174.699	4526.183	0.000 ^a^
	Yield	134,727.813	1	134,727.813	4086.606	0.000 ^a^
Ratio × Temp	C_PS_	0.707	1	0.707	18.319	0.000 ^a^
	Yield	356.780	1	356.780	10.822	0.002 ^a^
Ratio × Time	C_PS_	1.881	1	1.881	48.743	0.000 ^a^
	Yield	297.472	1	297.472	9.023	0.004 ^a^
Ratio × Cycle	C_PS_	8.690	1	8.690	225.144	0.000 ^a^
	Yield	1912.674	1	1912.674	58.016	0.000 ^a^
Temp × Time	C_PS_	0.108	1	0.108	2.806	0.100
	Yield	91.530	1	91.530	2.776	0.102
Temp × Cycle	C_PS_	7.273	1	7.273	188.436	0.000 ^a^
	Yield	5805.468	1	5805.468	176.093	0.000 ^a^
Time × Cycle	C_PS_	0.092	1	0.092	2.373	0.130
	Yield	87.173	1	87.173	2.644	0.110
Ratio × Temp × Time	C_PS_	0.001	1	0.001	0.024	0.877
	Yield	5.664	1	5.664	0.172	0.680
Ratio × Temp × Cycle	C_PS_	0.223	1	0.223	5.769	0.020 ^a^
	Yield	164.259	1	164.259	4.982	0.030 ^a^
Ratio × Time × Cycle	C_PS_	0.001	1	0.001	0.017	0.898
	Yield	14.367	1	14.367	0.436	0.512
Temp × Time × Cycle	C_PS_	0.333	1	0.333	8.629	0.005 ^a^
	Yield	222.513	1	222.513	6.749	0.012 ^a^
Ratio × Temp × Time × Cycle	C_PS_	0.057	1	0.057	1.475	0.230
	Yield	1.946	1	1.946	0.059	0.809

*SS*: sum of squares; *df*: degree of freedom; *MS*: mean square; C_PS_: polysaccharide concentration (mg/mL), Yield: extraction yield (mg/g). ^a^ Significant factors (*p* < 0.05).

**Table 4:** Polysaccharide concentration and extraction yield of *U. rigida* macroalgae under various conditions.

Ratio (*w*/*v*)	Temperature (°C)	Time (h)	Cycle	C_PS_ (mg/mL)	Yield (mg/g)
1:20	70	1	1	4.16 ± 0.38 ^d^	83.26 ± 7.58 ^f^
			2	0.78 ± 0.17 ^i^	15.58 ± 3.39 ^jk^
		2	1	4.88 ± 0.16 ^c^	97.57 ± 3.11 ^d^
			2	1.76 ± 0.21 ^g^	35.28 ± 4.13 ^gh^
	90	1	1	5.98 ± 0.11 ^b^	119.49 ± 2.11 ^c^
			2	1.41 ± 0.21 ^h^	28.29 ± 4.13 ^hi^
		2	1	7.28 ± 0.27 ^a^	145.56 ± 5.29 ^b^
			2	2.17 ± 0.15 ^f^	43.42 ± 2.89 ^g^
1:40	70	1	1	2.20 ± 0.20 ^f^	87.82 ± 8.10 ^ef^
			2	0.18 ± 0.03 ^k^	7.27 ± 1.30 ^k^
		2	1	2.37 ± 0.19 ^f^	94.91 ± 7.39 ^de^
			2	0.36 ± 0.07 ^jk^	14.56 ± 2.79 ^jk^
	90	1	1	3.49 ± 0.32 ^e^	139.41 ± 12.89 ^b^
			2	0.53 ± 0.08 ^ij^	21.12 ± 3.30 ^ij^
		2	1	3.98 ± 0.05 ^d^	159.23 ± 1.85 ^a^
			2	0.69 ± 0.20 ^i^	27.62 ± 7.98 ^hi^

Data in the same column with different superscripts are significantly different (*p* < 0.05). Data were calculated from triplicate experimental values ± standard deviation (SD).

**Table 5:** Total water-soluble polysaccharide extraction from *U. rigida* macroalgae.

Ratio (*w*/*v*)	Temperature (°C)	Time (h)	C_PS_ (mg/mL)	Yield (mg/g)
1:20	70	1	4.942 ± 0.46 ^d^	98.843 ± 9.12 ^f^
		2	6.643 ± 0.11 ^c^	132.846 ± 2.12 ^d^
	90	1	7.389 ± 0.16 ^b^	147.780 ± 3.18 ^c^
		2	9.449 ± 0.36 ^a^	188.970 ± 7.20 ^a^
1:40	70	1	2.377 ± 0.18 ^g^	95.089 ± 7.16 ^f^
		2	2.737 ± 0.18 ^f^	109.464 ± 7.21 ^e^
	90	1	4.014 ± 0.40 ^e^	160.534 ± 16.16 ^b^
		2	4.671 ± 0.17 ^d^	186.856 ± 6.63 ^a^

Data in the same column with different superscripts are significantly different (*p* < 0.05). Data were calculated from triplicate experimental values ± standard deviation (SD).

**Table 6:** Reducing sugar, total phenolic and flavonoid contents of polysaccharides extracted from *U. rigida* macroalgae under various conditions.

Ratio (*w/v*)	Temperature °C	Time (h)	Cycle	Reducing Sugar (mg/g)	TPC (mg GAE/g)	TFC (mg QE/g)
1:20	70	1	1	8.02 ± 3.32 ^a^	0.54 ± 0.10 ^bc^	0.22 ± 0.04 ^fgh^
			2	0.24 ± 0.15 ^bc^	ND	0.11 ± 0.05 ^hijk^
		2	1	6.50 ± 2.11 ^a^	0.60 ± 0.06 ^b^	0.21 ± 0.03 ^fghi^
			2	0.63 ± 0.30 ^bc^	ND	0.15 ± 0.03 ^ghij^
	90	1	1	5.76 ± 4.36 ^a^	0.52 ± 0.03 ^c^	0.34 ± 0.09 ^e^
			2	0.13 ± 0.13 ^bc^	ND	0.23 ± 0.04 ^fg^
		2	1	7.95 ± 2.36 ^a^	0.69 ± 0.02 ^a^	1.42 ± 0.16 ^a^
			2	0.37 ± 0.41 ^bc^	ND	0.28 ± 0.05 ^ef^
1:40	70	1	1	0.54 ± 0.26 ^bc^	0.18 ± 0.04 ^e^	0.64 ± 0.16 ^d^
			2	ND	ND	0.02 ± 0.01 ^k^
		2	1	0.82 ± 2.24 ^bc^	0.42 ± 0.03 ^d^	0.53 ± 0.03 ^d^
			2	ND	ND	0.03 ± 0.03 ^k^
	90	1	1	1.89 ± 2.24 ^b^	0.40 ± 0.12 ^d^	0.83 ± 0.12 ^c^
			2	ND	ND	0.08 ± 0.03 ^jk^
		2	1	6.58 ± 1.06 ^a^	0.60 ± 0.03 ^b^	0.99 ± 0.06 ^b^
			2	ND	ND	0.10 ± 0.03 ^ijk^

ND: Not detected; Data in the same column with different superscripts are significantly different (*p* < 0.05). Data were calculated from triplicate experimental values ± standard deviation (SD).

**Table 7:** Antioxidant activity of polysaccharides extracted from *U. rigida* macroalgae under various conditions.

Ratio (*w/v*)	Temperature °C	Time (h)	Cycle	DPPH (mg AAE/g)	ABTS (mg AAE/g)	FRAP (mg AAE/g)
1:20	70	1	1	6.47 ± 2.49 ^abcd^	0.87 ± 0.07 ^a^	0.26 ± 0.01 ^c^
			2	6.60 ± 2.42 ^abcd^	0.29 ± 0.04 ^c^	0.08 ± 0.01 ^e^
		2	1	8.76 ± 2.62 ^ab^	0.94 ± 0.13 ^a^	0.27 ± 0.00 ^c^
			2	6.84 ± 2.29 ^abcd^	0.27 ± 0.10 ^c^	0.17 ± 0.01 ^d^
	90	1	1	7.84 ± 1.86 ^abc^	0.78 ± 0.03 ^a^	0.24 ± 0.02 ^c^
			2	5.27 ± 2.86 ^bcde^	0.27 ± 0.08 ^c^	0.17 ± 0.01 ^d^
		2	1	4.10 ± 2.01 ^cdef^	0.79 ± 0.05 ^a^	0.27 ± 0.02 ^c^
			2	5.17 ± 0.86 ^bcde^	0.22 ± 0.09 ^c^	0.18 ± 0.01 ^d^
1:40	70	1	1	10.49 ± 3.56 ^a^	0.57 ± 0.19 ^b^	0.40 ± 0.04 ^b^
			2	0.05 ± 0.04 ^fg^	0.27 ± 0.09 ^c^	0.25 ± 0.01 ^c^
		2	1	1.37 ± 0.72 ^efg^	0.78 ± 0.13 ^a^	0.48 ± 0.03 ^a^
			2	0.02 ± 0.02 ^fg^	0.11 ± 0.08 ^c^	0.25 ± 0.01 ^c^
	90	1	1	ND	0.56 ± 0.13 ^b^	0.51 ± 0.10 ^a^
			2	0.03 ± 0.02 ^fg^	0.11 ± 0.22 ^c^	0.26 ± 0.01 ^c^
		2	1	2.39 ± 7.21 ^defg^	0.60 ± 0.10 ^b^	0.52 ± 0.02 ^a^
			2	0.09 ± 0.02 ^fg^	0.29 ± 0.16 ^c^	0.29 ± 0.01 ^c^

ND: Not detected; Data in the same column with different superscripts are significantly different (*p* < 0.05). Data were calculated from triplicate experimental values ± standard deviation (SD).

**Table 8:** Monosaccharide contents (in mg/g) from freeze-dried polysaccharides extracted and acid hydrolysis of freeze-dried polysaccharides from *U. rigida*.

Composition	Polysaccharide	Acid Hydrolysis of Polysaccharide
Rhamnose	ND	78.97 ± 2.71
Arabinose	ND	0.33 ± 0.02
Galactose	ND	2.81 ± 0.40
Glucose	0.11 ± 0.10	5.24 ± 0.28
Xylose	ND	12.11 ± 0.07
Fructose	ND	ND

ND: Not detected; Data were calculated from triplicate experimental values ± standard deviation (SD).

**Table 9:** Polysaccharide extraction yield for various algal species and conditions.

Macroalgae	Condition	Yield (%)	Reference
*Sargassum polycystum*	Acidic water, 60 °C, 1 h	59.80	[39]
*Chlorella* sp.	Water, 80 °C, 3 h	9.62	[40]
*Rhodosorus* sp.	Water, 84 °C, 2 h	9.29	[9]
*Sargassum thunbergii*	Water, 97 °C, 3.50 h	7.53	[41]
*Grifola* *frondosa*	Water, 210 °C, 43.65 min	25.10	[42]
*U. intestinalis*	Acidic solvent, Ultrasonic-assisted 200 W, 86 °C	26.77	[43]
*U. rigida*	80% ethanol, 73 °C, 2 h 6 min	13.22	[44]
*U. pertusa*	80% ethanol, Ultrasonic-assisted 600 W, 43.63 min	41.91	[45]
*U. rigida* C. Agardh	Water, 90 °C, 2 h	18.90	This study

**Table 10:** Monosaccharide compositions from various *Ulva* spp. species.

*Ulva* Species	Source	Ara	Fuc	Fru	Gal	Glc	Man	Rha	Xyl	Ref
*U. australis*	New Zealand	ND	ND	ND	3.00	ND	ND	251.00	99.00	[57]
*U. fasciata*	Philippines	ND	ND	ND	0.42	16.59	ND.	33.62	4.89	[58]
*U. meridionalis*	Japan	ND	ND	ND	28.27	144.43	5.40	66.56	12.34	[60]
*U. ohnoi*	Japan	ND	ND	ND	1.20	89.43	0.17	58.48	21.72	[60]
*U. pertusa Kjellm*	China	ND	ND	ND	2.20	3.60	ND	10.90	4.40	[66]
*U. rigida*	Chile	0.7	0.1	1.4	11.70	183.10	ND	81.20	38.50	[59]
*U. rigida*	New Zealand	ND	ND	ND	<1.00	ND	ND	257.00	27.00	[57]
*Ulva* spp.	New Zealand	ND	ND	ND	8.00	ND	ND	201.00	73.00	[57]

Ara = arabinose, Fuc = fucose, Fru = fructose, Gal = galactose, Glc = glucose, Man = mannose, Rha = rhamnose and Xyl = Xylose; ND: Not detected.

## Data Availability

The original contributions presented in the study are included in the article and Supplementary Materials, further inquiries can be directed to the corresponding author.

## Supplementary Materials

The following supporting information can be downloaded at https://www.mdpi.com/article/10.3390/foods13111630/s1, Figure S1: Polysaccharide extraction from *U. rigida* macroalgae under various conditions. (a,b) C_PS_ and extraction yield at 1:20 (*w*/*v*) biomass–solvent ratio, respectively; (c,d) C_PS_ and extraction yield at 1:40 (*w*/*v*) biomass–solvent ratio, respectively; Figure S2: Polysaccharide extraction from *U. rigida* macroalgae under various conditions. (a,b) C_PS_ and extraction yield at 70 °C, respectively; (c,d) C_PS_ and extraction yield at 90 °C, respectively; Figure S3: Polysaccharide extraction from *U. rigida* macroalgae under various conditions. (a,b) C_PS_ and extraction yield at an extraction time of 1 h, respectively; (c,d) C_PS_ and extraction yield at an extraction time of 2 h, respectively; Table S1: Standard curves of six monosaccharide reference standards.
