# Supplementary material for: Extraction, Characterisation and Evaluation of Antioxidant and Probiotic Growth Potential of Water-Soluble Polysaccharides from Ulva rigida Macroalgae
Source: Foods. 2024 May 23;13(11):1630. doi: 10.3390/foods13111630 (PMC11171798; doi:10.3390/foods13111630)
Supplement: Supplementary file 1 [file foods-13-01630-s001.zip › foods-3015897-supplementary.pdf]

Extraction, Characterisation and Evaluation of Antioxidant and Probiotic Growth Potential of Water-Soluble Polysaccharides from *Ulva rigida* Macroalgae

Naraporn Phomkaivon [1], Preeyanut Pongponpai [2], Prapat Kosawatpat [3],

Bussaba Thongdang [3] and Wanida Pan-utai [4,*]

[1] Department of Food Chemistry and Physics, Institute of Food Research and Product Development, Kasetsart University, Bangkok, 10900, Thailand; ifrnpph@ku.ac.th
[2] Faculty of Science, Prince of Songkla University, Songkhla, 90110, Thailand; preeyanut1415@gmail.com
[3] Phetchaburi Coastal Aquaculture Research and Development Center, Coastal Aquaculture Research and Development Division, Department of Fisheries, Phetchaburi, 76100, Thailand; prapat1120@gmail.com (PK), nok.annann@gmail.com (BT)
[4] Department of Applied Microbiology, Institute of Food Research and Product Development, Kasetsart University, Bangkok, 10900, Thailand; ifrwdp@ku.ac.th
* Correspondence: ifrwdp@ku.ac.th (W. Pan-utai); Address: Department of Applied Microbiology, Institute of Food Research and Product Development, Kasetsart University, Chatuchak, Bangkok, Thailand

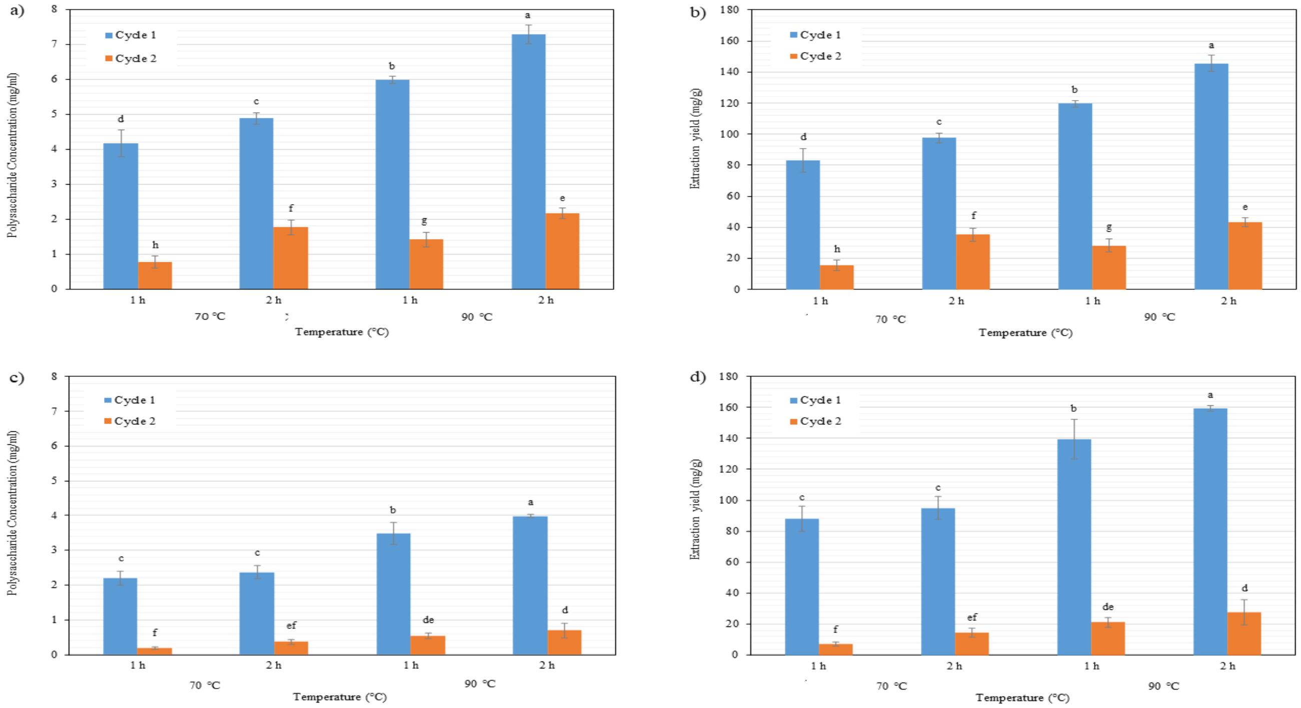

Figure S1. Polysaccharide extraction from *U. rigida* macroalgae under various conditions. a) and b) C_{PS} and extraction yield at 1:20 (w/v) biomass-solvent ratio respectively; c) and d) C_{PS} and extraction yield at 1:40 (w/v) biomass-solvent ratio respectively.

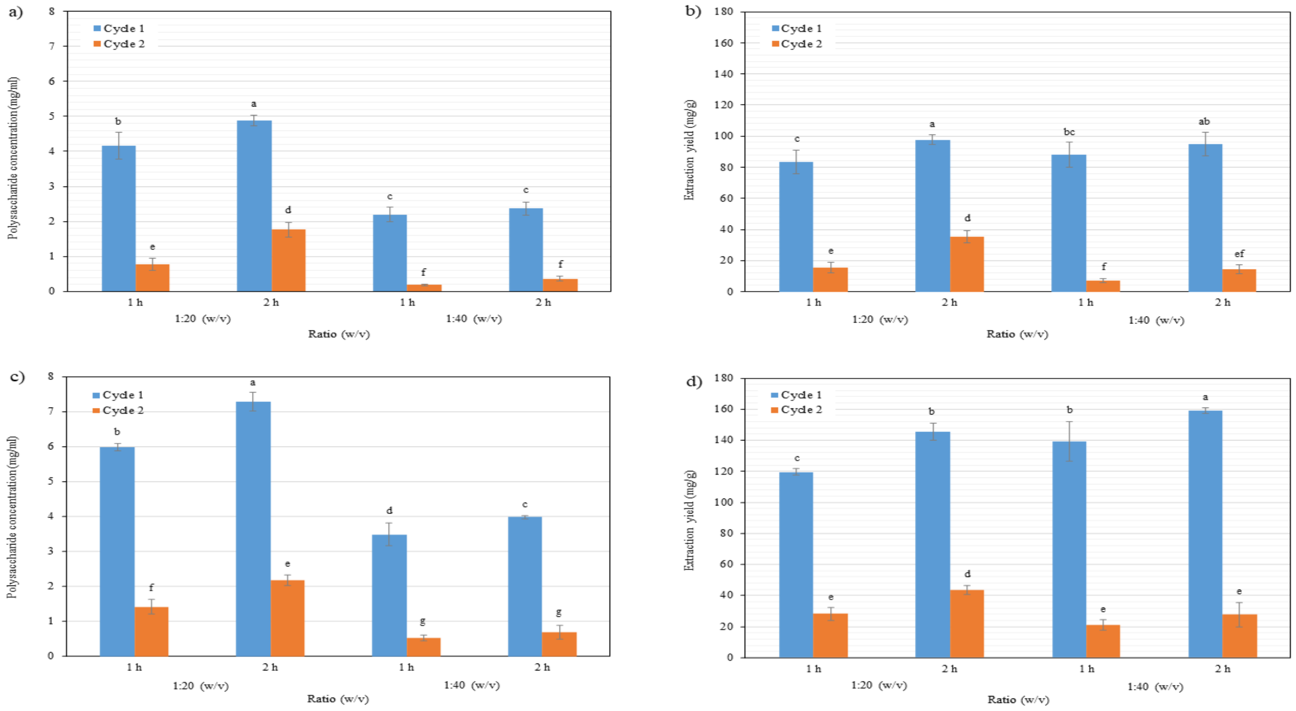

Figure S2. Polysaccharide extraction from *U. rigida* macroalgae under various conditions. a) and b) C_{PS} and extraction yield at 70°C respectively; c) and d) C_{PS} and extraction yield at 90°C respectively.

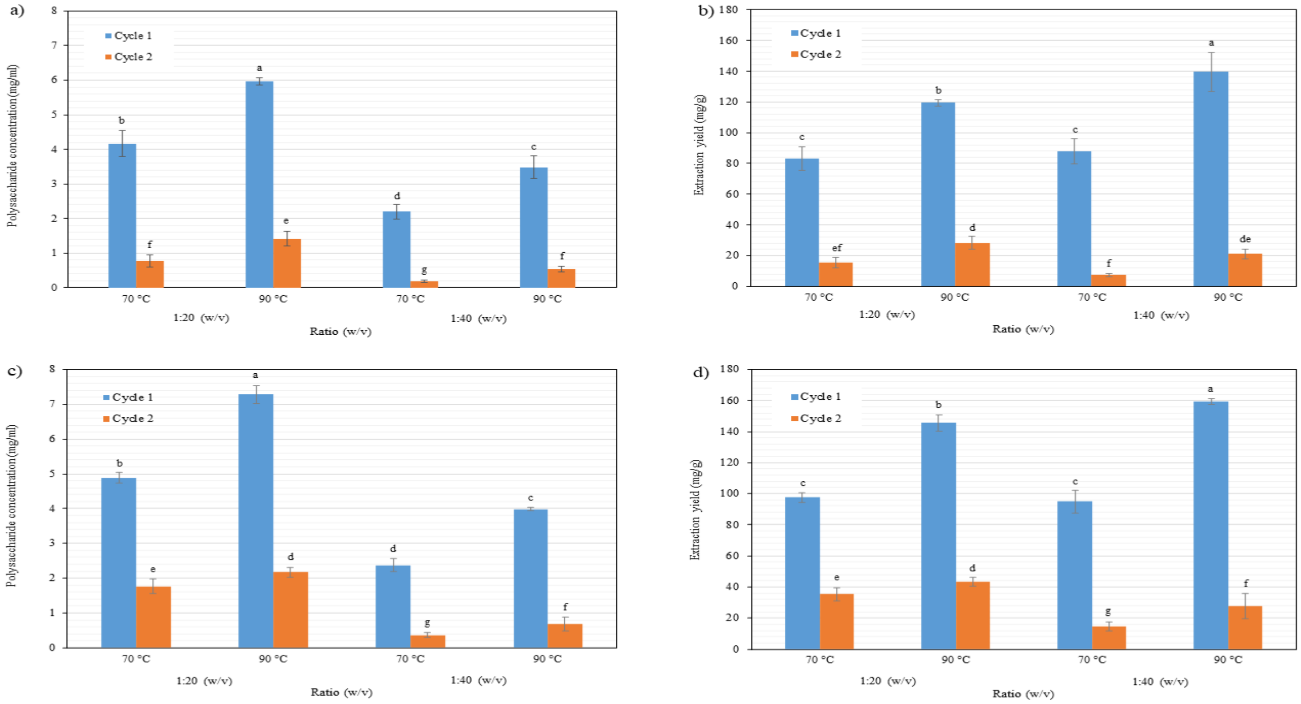

Figure S3. Polysaccharide extraction from *U. rigida* macroalgae under various conditions. a) and b) C_{PS} and extraction yield at an extraction time of 1 h respectively; c) and d) C_{PS} and extraction yield at an extraction time of 2 h respectively.

Table S1. Standard curves of six monosaccharide reference standards.

Composition	Retention time (min)	Regression equation	R^2
Rhamnose	6.507	y = 0.4561x + 3.0181	0.9917
Arabinose	7.054	y = 1.1304x + 0.1526	0.9990
Galactose	8.679	y = 1.1839x + 6.2965	0.9915
Glucose	9.599	y = 1.2369x + 4.6803	0.9959
Xylose	10.834	y = 1.2504x + 4.2593	0.9969
Fructose	12.217	y = 0.6287x + 2.98	0.9994
